# Epidermal Growth Factor Stimulates Extracellular-Signal Regulated Kinase Phosphorylation of a Novel Site on Cytoplasmic Dynein Intermediate Chain 2

**DOI:** 10.3390/ijms14023595

**Published:** 2013-02-07

**Authors:** Ashok K. Pullikuth, Aysun Ozdemir, Daviel Cardenas, Evangeline Bailey, Nicholas E. Sherman, K. Kevin Pfister, Andrew D. Catling

**Affiliations:** 1 Department of Pharmacology, School of Medicine, Louisiana State University Health Sciences Center, New Orleans, LA 70112, USA; E-Mails: apulliku@wakehealth.edu (A.K.P.); aozdem@lsuhsc.edu (A.O.); dcard1@lsuhsc.edu (D.C.); emckin@lsuhsc.edu (E.B.); 2 Department of Microbiology, Immunology and Parasitology, School of Medicine, Louisiana State University Health Sciences Center, New Orleans, LA 70112, USA; 3 W.M. Keck Biomedical Mass Spectrometry Laboratory, University of Virginia, Charlottesville, VA 22908, USA; E-Mail: nes3f@cms.mail.virginia.edu; 4 Department of Cell Biology, School of Medicine, University of Virginia, Charlottesville, VA 22908, USA; E-Mail: kkp9w@virginia.edu; 5 Stanley Scott Cancer Center, Louisiana State University Health Sciences Center, New Orleans, LA 70112, USA

**Keywords:** dynein, phosphorylation, ERK, dynactin

## Abstract

Extracellular-signal regulated kinase (ERK) signaling is required for a multitude of physiological and patho-physiological processes. However, the identities of the proteins that ERK phosphorylates to elicit these responses are incompletely known. Using an affinity purification methodology of general utility, here we identify cytoplasmic dynein intermediate chain *2* (DYNC1I-2, IC-2) as a novel substrate for ERK following epidermal growth factor receptor stimulation of fibroblasts. IC-2 is a subunit of cytoplasmic dynein, a minus-end directed motor protein necessary for transport of diverse cargos along microtubules. Emerging data support the hypothesis that post-translational modification regulates dynein but the signaling mechanisms used are currently unknown. We find that ERK phosphorylates IC-2 on a novel, highly conserved Serine residue proximal to the binding site for the p150^Glued^ subunit of the cargo adapter dynactin. Surprisingly, neither constitutive phosphorylation nor a phosphomimetic substitution of this Serine influences binding of p150^Glued^ to IC-2. These data suggest that ERK phosphorylation of IC-2 regulates dynein function through mechanisms other than its interaction with dynactin.

## 1. Introduction

The ERK cascade regulates a variety of processes including proliferation, survival, motility and differentiated cell function downstream of receptor tyrosine kinase stimulation. Many receptor tyrosine kinases, including the EGF receptor, are internalized by endocytosis following ligand-binding [1]. The original view was that internalization of the EGF receptor led to a termination of signaling activity and ultimately degradation of the activated receptor in lysosomes (reviewed in [2]). However, this notion was challenged by the demonstration that the majority of active EGF receptors and associated signaling molecules localize to early endosomes shortly after ligand stimulation [3–5], and that active EGF receptor interacts with Grb2 [6] and engages the major signaling pathways required for cell survival and mitogenesis after endocytosis [7,8]. Indeed, signaling from internalized active EGF and PDGF receptor signaling complexes is sufficient to stimulate cell survival [7] and proliferation [9], and long-range endosomal trafficking of activated neurotrophin receptors is essential for survival of neuronal cells [10]. In contrast, cell surface retention of the activated EGF receptor reduces the activity of some downstream signaling pathways, including ERK [11], and forced mislocalization of EGF receptor signaling to the periphery or perinuclear area alters nuclear signaling by ERK [12]. Together, these data are consistent with the overall hypothesis that intracellular trafficking of receptor tyrosine kinases on membrane bounded organelles is not simply a means to receptor degradation and signal termination but is essential for their signaling and physiological functions.

Diverse observations suggest that ERK regulates movement of membrane bounded organelles along microtubules. A fraction of ERK has long been known to associate with microtubules [13–15] and it is perhaps interesting to reflect that ERK was once known as microtubule-associated protein (MAP) kinase [16]. Melanosomes support activation of MEK (MAP kinase or ERK, kinase) and ERK on their cytoplasmic surface and melanosome movement along microtubules is blocked by MEK inhibitor in amphibians [17,18]. The mechanism by which ERK regulates melanosome movement is unknown, but Olofsson and colleagues have identified the motor protein dynein as a potential target for ERK during the formation of cytosolic lipid droplets [19], organelles that bud from the plasma membrane, contain microsomal proteins, and whose formation requires microtubules and motor proteins. Of particular relevance, knock-out of the ERK activator MEK1 displaces late endosomes to the periphery [20,21].

Here we identify cytoplasmic dynein intermediate chain 2 (DYNC1I-2 [22], IC-2) as a novel ERK substrate. We identify the major site of EGF-stimulated intermediate chain phosphorylation as Serine 81 and show that ERK activity is necessary and sufficient for its phosphorylation. Dynein intermediate chain 2 is robustly and dynamically phosphorylated in response to EGF stimulation. Given its proximity to phosphorylation sites shown to inhibit binding to the cargo adapter dynactin, we were surprised to find that binding to the p150^Glued^ subunit of dynactin was unaffected by Serine 81 phosphorylation. We hypothesize that ERK phosphorylation regulates dynein through mechanisms other than its interaction with dynactin.

## 2. Results

### 2.1. Identification of Potential ERK Substrates Using Phosphomotif-Specific Antisera

**Figure 1:**
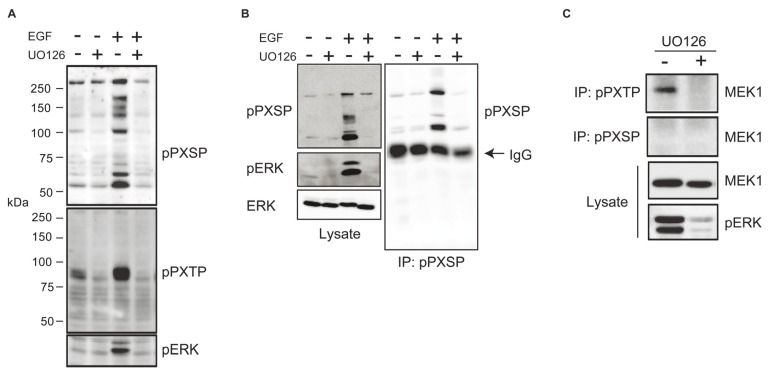
(**A**) Phospho-motif antisera recognize potential ERK substrates. Serum-starved cultures were pre-treated with or without the MEK inhibitor UO126 before stimulation with EGF for 10 min. Lysates were blotted with anti-phospho PXSP, anti-phospho PXTP or anti-phospho ERK antisera. (**B**), (**C**) Phospho-motif antisera immunoprecipitate known and potentially novel ERK substrates. Lysates prepared as in (**A**) were immunoprecipitated (IP) with anti-phospho PXSP (**B**) or anti-phospho PXTP (**C**) antisera. Immunoprecipitates were blotted with anti-phospho PXSP antiserum (**B**) or with antiserum to MEK1, a known ERK substrate (**C**).

ERK often phosphorylates substrate proteins on Serine and Threonine residues in PXSP and PXTP sequence motifs (where X is any amino acid) [23]. We first asked whether antisera with affinity for phospho PXSP and phospho PXTP motifs could blot proteins potentially phosphorylated directly by ERK in response to growth factor stimulation. Little phospho PXSP or phospho PXTP reactivity was seen in serum-deprived fibroblasts whereas a number of proteins were detected in EGF stimulated cells (Figure 1A). Anti-phospho PXSP and anti-phospho PXTP reactivity was inhibited by pre-treatment of cells with UO126, a MEK inhibitor (Figure 1A). A number of proteins were immunoprecipitated by anti-phospho PXSP antiserum from lysates of EGF stimulated cells and immunoprecipitation was blocked by prior treatment of cells with UO126 (Figure 1B). Similar data were obtained using the anti-phospho PXTP motif antiserum (data not shown). To extend these observations we asked if we could identify a known ERK substrate using this affinity purification approach. MEK1 is phosphorylated on Threonines 292 and 386 within PXTP motifs in response to ERK activation [24–26] and it was immunoprecipitated with the anti-phospho PXTP (but not anti-phospho PXSP) antiserum when ERK was active (Figure 1C). These data demonstrate that putative ERK substrates can be immunopurified with the phosphomotif-specific antisera.

### 2.2. Identification of Dynein Intermediate Chain 2 as a Potential ERK Substrate

**Figure 2:**
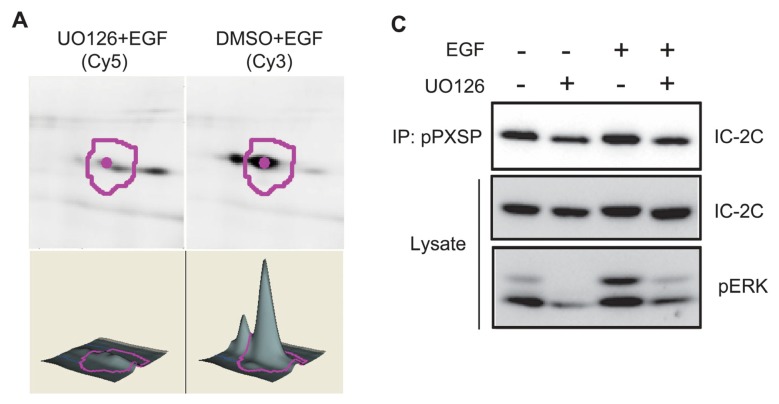

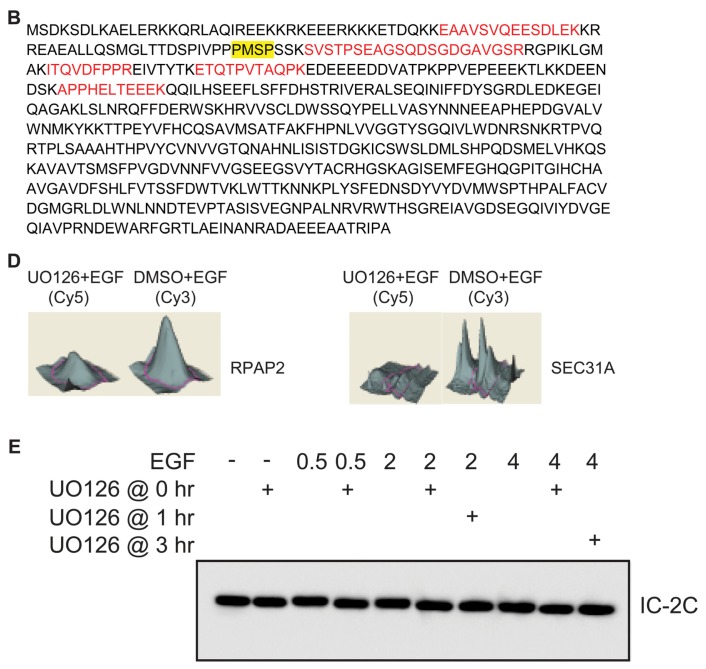
(**A**) 2D DIGE identification of a candidate ERK substrate. Proteins phosphorylated on PXSP motifs were immunoprecipitated from cultures treated with EGF with or without prior treatment with the MEK inhibitor UO126. Immune complexes were labeled with Cy5 or Cy3 respectively, combined and resolved on a single 2D gel. Proteins whose immunoprecipitation by anti-phospho PXSP antiserum was substantially inhibited by pre-treatment with UO126 (*i.e.*, Cy3 fluorescence > Cy5 fluorescence) were picked for identification. Shown are the Cy3 and Cy5 fluorescence images and quantitation for the protein subsequently identified as dynein intermediate chain 2C; (**B**) Identification of dynein intermediate chain 2C (IC-2C). Following digestion with Trypsin, the indicated peptides (red) were identified by MS/MS (see Methods). The highlighted sequence identifies the PXSP motif presumably recognized by the anti-phospho PXSP antiserum; (**C**) Independent identification of dynein intermediate chain in phospho PXSP motif immunoprecipitates. Phospho PXSP immunoprecipitates were blotted with monoclonal antibody against dynein intermediate chains (top). Pre-treatment with UO126 inhibits both ERK phosphorylation (bottom) and immunoprecipitation of dynein intermediate chain. Note the EGF-stimulated, UO126-sensitive mobility shift of dynein intermediate chain in the lysates (middle panel); (**D**) Tentative identification of potential ERK substrates in phospho PXTP immunoprecipitates; see (**A**); (**E**) Dynamic phosphorylation of dynein intermediate chain following EGF stimulation. Dynein intermediate chain is mobility shifted following EGF stimulation for 30 min. or 2 hr (compare lanes 1, 3 and 5). As expected, mobility shifting can be inhibited by pre-treatment with UO126 (lanes 2, 4 and 6) or by adding UO126 1 hr post-EGF stimulation.

To identify new ERK substrates we treated cells with EGF for 30 min. with or without prior treatment with UO126. Anti-phospho PXSP immunoprecipitates were made from each lysate, labeled with Cy5 and Cy3 dye respectively, combined and resolved on a single 2D gel. By comparing fluorescence intensities between Cy3 and Cy5 channels a number of proteins whose apparent abundance was substantially decreased in immunoprecipitates from UO126-treated cells were identified. One spot much less abundant in the UO126-treated sample (Figure 2A) was picked, digested with Trypsin and identified by mass spectrometry with a high degree of confidence as the cytoplasmic dynein intermediate chain IC-2 (Figure 2B). To verify this identification, we blotted independent anti-phospho PXSP immunoprecipitates with dynein intermediate chain monoclonal antibody 74.1 [27]. Dynein intermediate chain was present in anti-phospho PXSP immunoprecipitates from EGF-stimulated WT cells, and the amount of dynein in the immunoprecipitate was reduced by pretreatment of cultures with UO126 (Figure 2C). To confirm the general utility of this approach, we performed similar experiments with anti-phospho PXTP antiserum. Two proteins whose apparent abundance was substantially decreased in immunoprecipitates from UO126-treated cells (Figure 2D) were tentatively identified by mass spectrometry as SEC31A and RNA polymerase II-associated protein 2 (RPAP2, data not shown). These data are evidence that this affinity purification approach can identify potential ERK substrates with novel functions.

**Figure 3:**
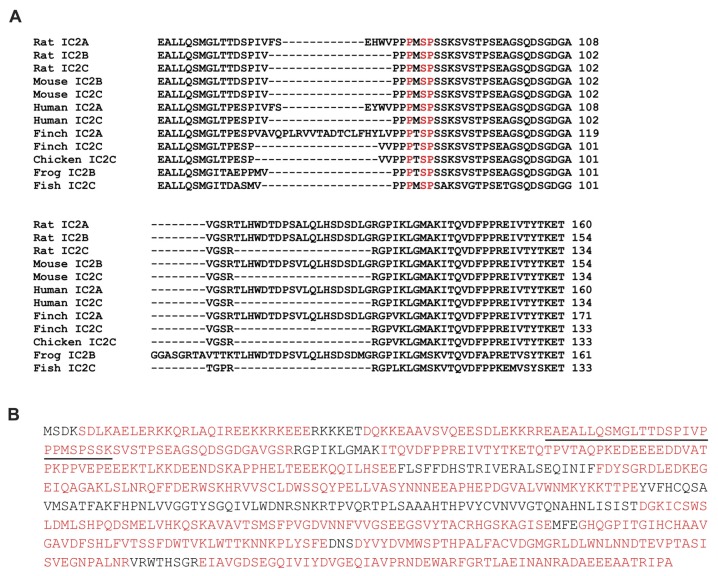

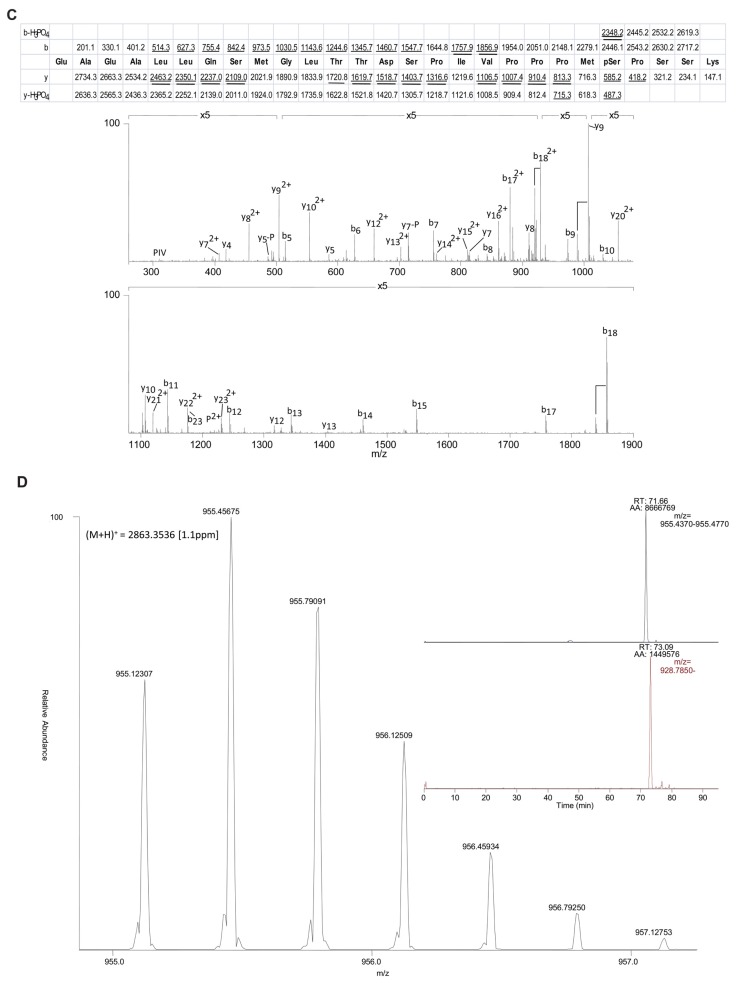
(**A**) Serine 81 lies within a PXSP (highlighted in red) motif conserved in vertebrates. Aligned are partial primary sequences from rat (*Rattus norvegicus*), mouse (*Mus musculus*), human (*Homo sapiens*), zebra finch (*Taeniopygia guttata*), chicken (*Gallus gallus*), Western clawed frog (*Xenopus tropicalis*) and zebrafish (*Danio rerio*); (**B**) IC-2C was purified from EGF stimulated cultures and subject to digestion with Trypsin, endoproteinase Asp-N or endoproteinase Glu-C. Residues identified by mass spectrometry are shown in red (~78% sequence coverage). The tryptic peptide containing Serine 81 is underlined; (**C**) MS/MS spectrum for the tryptic peptide containing Serine 81. The theoretical *b*- and *y*-ions are shown and those found experimentally are underlined; a double underline indicates that the doubly charged ion was also seen. These ions are also shown on the spectrum along with several brackets indicating prominent water losses. The spectrum is magnified 5X except for the *y*_9_, *y*_9_^2+^ and *b*_18_^2+^ ions which are extremely intense proline directed fragments; (**D**) The parent *m/z* for the S81 phosphorylated peptide producing a M+H^+^ of 2863.3536 (1.1 ppm). This particular peptide was seen in four forms due to the presence of two Methionine residues. Selected ion chromatograms (inset) were used to estimate the ratio of phosphorylated S81 peptide to non-phosphorylated S81 peptide in control and EGF-stimulated samples following either Trypsin or Asp-N digestion (see Table 2).

There are several isoforms of the vertebrate cytoplasmic dynein intermediate chain, the product of two genes and alternative splicing [28–31]. However, it is most likely that IC-2C is the isoform present in these cells, as all of the fibroblastic cell lines examined to date express only this isoform [28,30,32,33]. In addition our mass spectrometry analyses failed to detect the peptides that would unambiguously identify the other IC-2 isoforms, IC-2A and IC-2B, in the immunoprecipitates (shown in Figure 3A). This is consistent with observations that the only cultured cells in which IC-2B was found were neuronal cells, and that IC-2A was found only in brain tissue [28,29,34–36]. Similarly, no peptides from intermediate chain 1 were observed in our mass spectra (data not shown). This is consistent with the observations that IC-1 isoforms have only been found in cultured primary neurons or tissue from brain, testis, or ovary [28–32,37].

The migration of the IC-2C band shifted to a slightly higher apparent molecular weight in response to EGF stimulation, and this mobility shift was blocked by pre-treatment with UO126 (Figure 2E). While not definitive, UO126-sensitive mobility shifting is consistent with ERK stimulating phosphorylation of IC-2C. In repeated time course experiments we found that mobility shifting of IC-2C was maintained for at least 2 hrs post EGF-stimulation (Figure 2E; data not shown). Interestingly, mobility shifting at the 2 hr time point could be blocked by UO126 given either before or significantly after EGF stimulation (Figure 2E), indicating that initial MEK-dependent phosphorylation of IC-2C is not indefinitely stable, but rather that dynamic MEK-dependent dephosphorylation/phosphorylation of IC-2C occurs post EGF-stimulation. We conclude that IC-2C is a MEK-dependent substrate phosphorylated on ERK consensus (PXSP) motifs following EGF stimulation.

### 2.3. EGF Stimulates Phosphorylation of Serine 81 of Dynein Intermediate Chain 2C

IC-2C contains a single PXSP motif (PMSP, residues 79–82; Figure 2B) conserved in available vertebrate IC-1 and IC-2 sequences (Figure 3A). We sought to determine if EGF stimulates phosphorylation of Serine 81 within this motif. IC-2C was immunoprecipitated with monoclonal antibody 74.1 under stringent conditions and digested with Trypsin, endoproteinase Glu-C or endoproteinase Asp-N. Resulting peptides were analyzed by LC/MS and MS/MS as described in Methods. Digestion with Trypsin, Glu-C and Asp-N yielded 78% amino acid coverage of IC-2C from EGF-stimulated cells (Figure 3B) and similar coverage in the unstimulated sample (data not shown). Serine 81 was identified as the predominant site of phosphorylation in EGF stimulated cells as determined by the ratio of the abundance of phosphorylated to non-phosphorylated species (Table 1); five other phosphorylation sites were identified at much lower abundance (Table 1). Figure 3C shows the MS/MS spectrum for the tryptic peptide containing Serine 81 (underlined in Figure 3B). Selected ion chromatograms (Figure 3D, inset) were used to estimate the ratio of phosphorylated S81 peptide to non-phosphorylated S81 peptide (+P/−P) in control and EGF-stimulated samples following either Trypsin or Asp-N digestion (Table 2). Serine 81 phosphorylation increased 5.5–6 fold following 30 min. EGF stimulation (Table 2).

### 2.4. ERK Activity Is Necessary and Sufficient to Stimulate Serine 81 Phosphorylation of Dynein Intermediate Chain 2C

**Figure 4:**
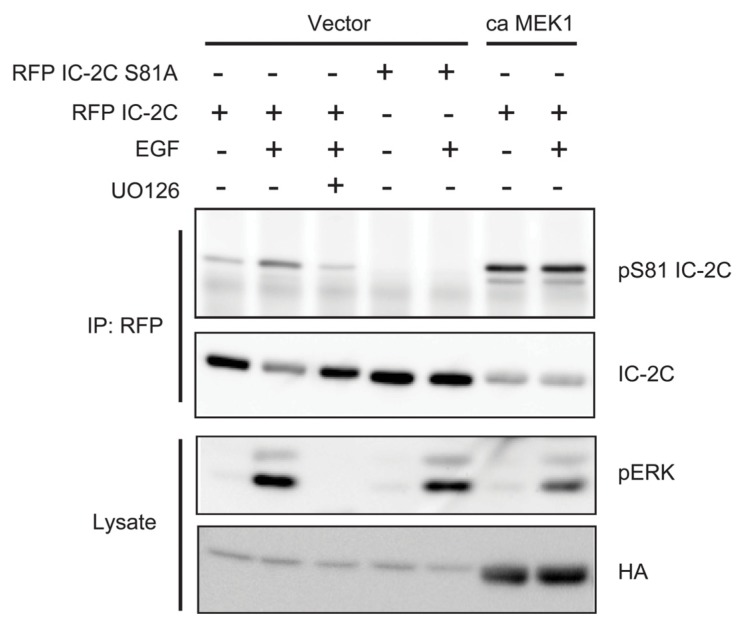
MEK activity is necessary and sufficient to stimulate phosphorylation of IC-2C Serine 81. Cells were transiently transfected with the indicated mRFP-tagged IC-2C construct with or without HA-tagged constitutively active MEK1. Cultures were serum-starved before treatment with UO126 and EGF as indicated. Anti-mRFP immunoprecipitates were blotted with anti-phospho Serine 81 IC-2C antiserum, stripped, and blotted with anti-dynein intermediate chain antibody. EGF stimulates phosphorylation of wild type IC-2C on Serine 81, and this phosphorylation is substantially inhibited by prior treatment with UO126. Expression of constitutively active MEK1 renders phosphorylation independent of EGF stimulation. Note that the apparent differences in IC-2C loading signal result from incomplete removal of the phospho-S81 antiserum.

To test whether ERK activity is necessary and sufficient for EGF stimulated IC-2C phosphorylation, we co-transfected mRFP-tagged IC-2C with either empty vector or constitutively active (ca) MEK1 in which the sites of activating phosphorylation, Serines 218 and 222, are mutated to phosphomimetic aspartate residues [25]. Cells were deprived of serum, treated with or without UO126 and stimulated with EGF for 10 min. Anti-phospho Serine 81 antibody [38] was used to detect mRFP-IC-2C phosphorylated on Serine 81. Serine 81 of mRFP-IC-2C was phosphorylated at low but detectable levels in unstimulated cells and induced by EGF (Figure 4). Pre-treatment with UO126 prevented EGF-stimulated Serine 81 phosphorylation (Figure 4). In contrast, co-transfection with constitutively active (ca) MEK1 induced robust Serine 81 phosphorylation in the absence of EGF stimulation (Figure 4). Mutation of Serine 81 to alanine eliminated reactivity with the anti-phospho Serine 81 antibody, confirming its specificity. These observations indicate that MEK and by extension ERK activity are necessary and sufficient for phosphorylation of IC-2C on Serine 81. In a companion paper we verified direct phosphorylation of recombinant IC-2C with recombinant active ERK1 and Mg^2+^/ATP *in vitro*. ERK1 phosphorylated IC-2C on Serine 81 in a time-dependent manner as measured by blotting with the anti-phospho Serine 81 antiserum [38].

### 2.5. Phosphorylation Does Not Regulate Binding of Dynein Intermediate Chain to p150^Glued^

**Figure 5:**
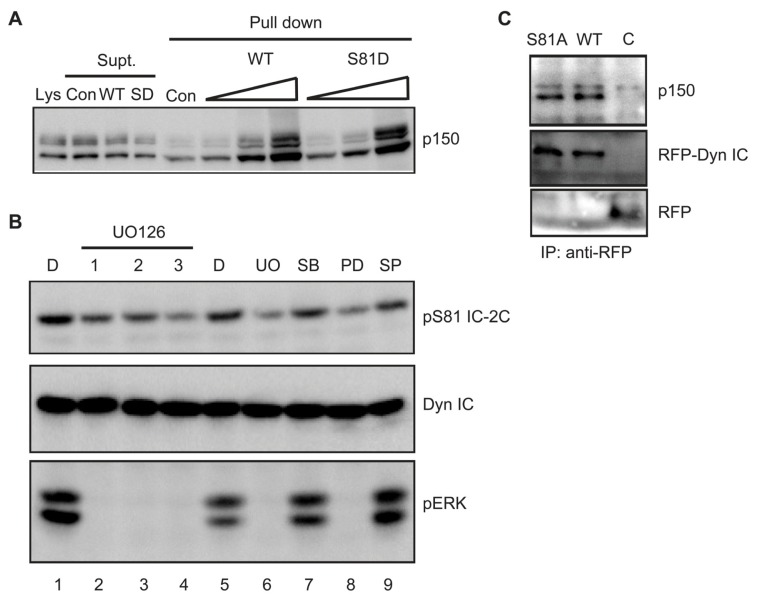
(**A**) Mimicking S81 phosphorylation has no effect on the ability of recombinant IC-2C to bind p150^Glued^. Increasing amounts (0.5–12.5 μg) of recombinant polyhistidine-tagged wild type (WT) and S81D IC-2C were incubated with brain lysate and bound proteins recovered by pull down and imidazole elution from Nickel agarose beads; empty Nickel agarose beads (Con) served as a negative control. 1% of the starting lysate is shown (Lys) together with 1% of the supernatants following control bead pull down (Con) and following pull down with 12.5 μg of either wild type or S81D IC-2C. Multiple p150 bands are routinely observed when dynactin from brain lysates is analyzed [36]. They are known to be products of alternative splicing [39] and/or phosphorylation [40,41]; (**B**) A375 melanoma cells exhibit constitutive ERK-dependent phosphorylation of IC-2C. A375 cells were serum deprived overnight before treatment with either DMSO vehicle (D) for 3 h, or UO126 for 1–3 h (lanes 1–4). Phosphorylation of IC-2C on S81 is lost in a time-dependent manner. Pre-incubation with a distinct MEK inhibitor (PD98058) similarly inhibited IC-2C phosphorylation (lane 8) whereas inhibitors of p38 (SB203580; lane 7) and JNK (SP600125; lane 9) were without substantial effect; (**C**) Phosphorylation of S81 is not required for p150^Glued^ binding. A375 cells were transiently transfected with mRFP-tagged IC-2C constructs, and P2 membrane fractions were immunoprecipitated using anti-RFP antiserum. Similar amounts of p150^Glued^ were co-immunoprecipitated with wild type and S81A IC-2C.

Dynein engages many cargoes through an interaction between its intermediate chains and the p150^Glued^ subunit of the dynactin cargo adapter complex [42]. Since Serine 81 is proximal to sequences mediating the intermediate chain-dynactin interaction, we asked whether phosphorylation alters p150^Glued^ binding. First we asked if mimicking S81 phosphorylation modulates binding of p150^Glued^ to recombinant IC-2C protein. Bacterially expressed (non-phosphorylated) wild type and S81D IC-2C proteins were equally able to bind p150^Glued^ in these pull-down assays (Figure 5A). Second, we asked if phosphorylation *in situ* would regulate co-immunoprecipitation of p150^Glued^ with IC-2C. A375 melanoma cells express an oncogenic form of B-Raf that causes constitutive activation of ERK [43]. We found that endogenous dynein intermediate chain(s) are constitutively phosphorylated on Serine 81 in A375 cells, and that phosphorylation can be substantially reduced by treating cultures with the MEK inhibitors UO126 or PD90859 but not with inhibitors of JNK or p38 MAPK (Figure 5B), confirming that phosphorylation is largely mediated by ERK. p150^Glued^ co-immunoprecipitated equally well with mRFP-tagged wild type or S81A IC-2C from transiently transfected A375 melanoma cells, suggesting that Serine 81 phosphorylation is without effect on the IC-2C-dynactin interaction (Figure 5C). These data suggest that phosphorylation at S81 does not markedly alter the affinity of the intermediate chain for p150^Glued^.

### 2.6. A Portion of Phospho Serine 81 Dynein Intermediate Chain Fractionates with Membrane Bound Organelles

**Figure 6:**
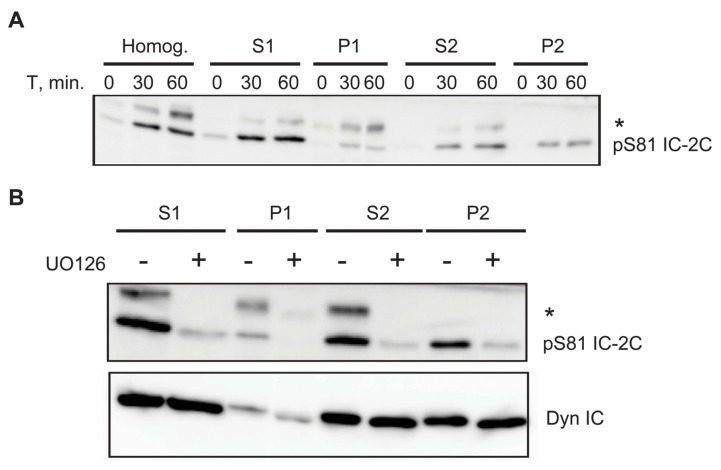
(**A**) Phospho S81 IC-2C fractionates with membrane bound organelles. Serum-starved REF cells were left untreated or stimulated with EGF for 30 or 60 min. before homogenization. Extracts were fractionated as described in Methods and normalized fractions were probed for phospho IC-2C. Approximately 40% of Serine 81-phosphorylated IC-2C is found in the high speed pellet (P2) after EGF stimulation. * Denotes a non-specific protein that does not blot or immunoprecipitate with anti-dynein intermediate chain antibody; (**B**) MEK activity and S81 IC-2C phosphorylation are dispensable for fractionation of IC-2C in the high speed pellet. Cells were fractionated as described in (**a**) except cultures were pre-treated with or without the MEK inhibitor UO126. UO126 substantially inhibits S81 phosphorylation but does not prevent IC-2C from fractionating in P2. *, See (**A**).

Dynein is instrumental in the intracellular trafficking of membrane-bound cargos [42]. To ask whether Serine 81 phosphorylated IC-2C associates with membrane bound organelles we performed subcellular fractionation experiments. Serum-deprived cells were treated with or without EGF for time periods up to 60 min. before homogenization and fractionation as described [44,45]. We found that approximately 40% of IC-2C phosphorylated on Serine 81 was recovered in a high speed postnuclear pellet (P2; Figure 6A). The remainder of the phospho-intermediate chain was largely found in the high speed supernatant (S2; ~40%) with a small portion fractionating in the low speed pellet (P1; Figure 6A) containing cell ghosts and nuclei. Pre-treatment with the MEK inhibitor UO126 substantially inhibits Serine 81 phosphorylation without detectably altering the level of IC-2C recovered in P2 (Figure 6B).

## 3. Discussion

Intracellular trafficking of receptor tyrosine kinases involves the actions of at least three motor protein complexes: the kinesin and myosin families and cytoplasmic dynein (reviewed in [46]). The dynein complex is a minus-end directed microtubule motor that traffics membrane associated cargo (including Golgi apparatus, lysosomes, and endosomes) towards the microtubule organizing center in the perinuclear region of the cell [47–49]. Depolymerization of microtubules or inhibition of dynein function attenuates retrograde delivery of Trk receptors activated in axons to the cell body, and inhibits cell survival [10]. Similarly, genetic interactions between dynein and EGF receptor in *Drosophila* support the idea that dynein is important for EGF receptor function in eye development [50], and indeed inhibition of dynein function inhibits the movement of EGF-containing early endosomes towards the perinuclear region of HeLa cells [51] and redistributes late endosomes to the cell periphery [52]. Furthermore, dynein activity is important for the sorting of internalized EGF receptor, destined for degradation, from the recycling transferrin receptor [51]. From these experiments it is clear that dynein regulates tyrosine kinase receptor fate. Importantly, it is now becoming apparent that receptor signaling regulates dynein function. Thus, dynein-dependent retrograde trafficking of the TrkB receptor is dependent upon receptor kinase activity [10], and trafficking of TrkA signaling endosomes is promoted by NGF stimulation [37]. These elegant studies demonstrate essential roles for the dynein/microtubule system in the trafficking of tyrosine kinase receptor signaling endosomes, and a reciprocal role for the receptors in regulating dynein-mediated trafficking. However, it is not known which receptor signaling pathways regulate receptor movement, and how these regulatory influences modify dynein function.

Each cytoplasmic dynein complex has two heavy chains that contain a motor domain encoding ATPase units and microtubule binding elements, and a smaller *N*-terminal domain that encodes the cargo binding function of the complex. Most of the dynein cargo binding subunits bind to the base of the molecule. Thus, the intermediate and light intermediate chains bind as dimers to the base of the heavy chain, while the light chain dimers bind the intermediate chains. While it is clear that the dynein heavy chain functions as an ATP-dependent microtubule motor [48,49], the function of the intermediate and light chains is only now coming into focus. There are two dynein intermediate chain genes, but alternative splicing yields at least six intermediate chain isoforms [37] that are differentially expressed according to tissue and/or physiological state [34,53]. This diversity of expression is proposed to contribute to cargo selectivity, and indeed dynein complexes containing distinct intermediate chains are differentially localized in optic nerve axons [36] and specific intermediate chain isoforms transport TrkB signaling endosomes [44]. However, some cell types, including fibroblasts and glia, express only intermediate chain 2 splice form C [29,30,33,37]. In these cases, cargo binding specificity must result in part from differential regulation of the common intermediate chain. Intermediate chains are known to be phosphorylated [27,37], and phosphorylation can be regulated by receptor stimulation [34,54]. Relatively little is known regarding the identity or regulatory role of such phosphorylation sites.

### 3.1. Identification of Dynein Intermediate Chain 2C as an ERK Substrate

To identify new targets for ERK phosphorylation in the context of EGF stimulation we used Cell Signaling Technology, Inc.’s PTMScan technology. This approach uses antibodies to phosphorylated kinase consensus motifs as a means to identify substrates. Using antisera that recognize phosphorylated Serine or Threonine residues in consensus motifs for ERK phosphorylation (PXSP and PXTP, where X is any amino acid [23]), initial experiments (Figure 1) demonstrated that we could blot and immunoprecipitate candidate and known ERK substrates, and importantly, that antibody reactivity was largely dependent upon MEK activity. Since other proline-directed kinases (e.g., cyclin-dependent kinases) may utilize these phosphorylation motifs, we combined anti-phospho PXSP immunoprecipitation with prior treatment of EGF-stimulated cultures with or without the MEK inhibitor UO126. Immunoprecipitates from UO126- and vehicle-treated cells were individually labeled with Cy5 and Cy3 respectively before being combined and resolved on a single 2D gel. By comparing fluorescence intensities between Cy3 and Cy5 channels, a number of proteins whose apparent abundance was substantially decreased in UO126-treated cells were identified. One such MEK-dependent candidate was identified by mass spectrometry with a high degree of confidence as dynein intermediate chain 2C (Figure 2).

### 3.2. Dynein Intermediate Chain 2C Is Phosphorylated on Serine 81

Inspection of the IC-2C sequence reveals a single PXSP motif (PMSP) encompassing Serine 81 (Figure 2) that is invariant in available vertebrates IC-2 and IC-1 sequences (Figure 3A). Using a combination of Trypsin, endoproteinase AspN and endoproteinase GluC digests combined with mass spectrometry, we identified Serine 81 as the predominant site of EGF stimulated phosphorylation. Calculations were performed to determine changes in phosphorylation between samples. Serine 81 phosphorylation rises ~6 fold following 30 min. EGF stimulation, and then returns to baseline by 4 h EGF stimulation (Table 2 and data not shown). These data are in good agreement with data obtained using the phospho S81 IC-2C antiserum (Figure 4).

In addition to the predominant site of phosphorylation at Serine 81, we identified five additional minor (low +P/−P ratio) phosphorylation sites in mouse IC-2C: Serine 51, Threonine 89, Serine 95, Serine 98 and Threonine 154 (Table 1). The weakness of these sites and in cases their overlapping chromatography precluded definitive conclusions regarding their regulation by EGF. Threonine 89 was previously identified as a site of phosphorylation in mitotic cells [33]. Of potential interest, Serine 51 is flanked *N*- and *C*-terminal by a number of negatively charged residues making it a candidate for phosphorylation by casein kinase II [55]. Casein kinase II was previously shown to associate with and *in vitro* phosphorylate dynein intermediate chain on an unknown site(s) [56] but to our knowledge it is not known if this enzyme phosphorylates dynein in cells. Serine 51 also resides within a potential consensus for phosphorylation by Casein kinase I [55] and Casein kinase I was recently shown to phosphorylate *Xenopus* dynein intermediate chain and stimulate minus-end directed movement of melanophores [57]. Serines 95 and 98 are both found within SXD motifs; such motifs have been found to be substrates for Ca^2+^/calmodulin-dependent kinase II in some proteins [42,58,59]. Further work is necessary to determine if these sites and/or kinases are physiologic regulators of dynein. Notably, we did not detect phosphorylation of Serine 84 [32] which was previously implicated in Golgi distribution [32].

### 3.3. ERK Activity Is Necessary and Sufficient to Phosphorylate Serine 81

Using MEK inhibitor and a constitutively active form of MEK1 we determined that MEK activity is necessary and sufficient for phosphorylation of IC-2C in fibroblasts (Figure 4). Similarly, intermediate chains are constitutively phosphorylated in a MEK-dependent manner in A375 melanoma cells endogenously expressing an oncogenic form of B-Raf (Figure 5B). *In vitro* reconstitution assays verified that ERK directly phosphorylates IC-2C on Serine 81 [38]. Although our data make clear that ERK is largely responsible for phosphorylating IC-2C on Serine 81 after EGF stimulation, two observations warrant further consideration. First, while pre-treatment with UO126 inhibits EGF-stimulated Serine 81 phosphorylation as expected, we were surprised to find that UO126 added significantly after EGF stimulation also inhibited IC-2C phosphorylation. One possibility is that initial IC-2C phosphorylation is unstable, and that both dephosphorylation and MEK-dependent phosphorylation of IC-2C occur over the time course. However, we cannot rule out an alternate mechanism whereby MEK or ERK inhibit a phosphatase that dephosphorylates IC-2C. Adding UO126 after EGF stimulation would be predicted to elevate this phosphatase activity and cause the observed dephosphorylation of IC-2C.

Second, it should be noted that neither serum-starvation nor UO126 completely eliminate reactivity with the phospho Serine 81 antibody in fibroblasts, while mutation of Serine 81 to Alanine does. Similarly, MEK inhibitors do not entirely eliminate S81 phosphorylation of endogenous intermediate chain(s) in A375 melanoma cells. These observations indicate that some intermediate chain is likely phosphorylated on Serine 81 by kinases distinct from ERK and/or resides in compartments not accessed by the MEK inhibitors.

### 3.4. Association of Phospho-S81 IC-2C with Membrane Bounded Organelles

We postulated that IC S81 phosphorylation triggered by EGF stimulation of ERK might be important for the recruitment of dynein to EGF receptor signaling endosomes. This is consistent with the observations that MEK1 is required for perinuclear localization of late endosomes containing EGFR [20] and that S81 phosphorylation triggered by neurotrophin binding to Trk receptors is necessary for dynein binding to Trk containing signaling endosomes but not mitochondria [38]. In efforts to test this hypothesis we found that pre-treatment of cells with UO126 largely eliminated EGF-stimulated S81 phosphorylation and had no discernible effect on the fractionation pattern of total IC-2C. This observation was consistent with the lack of correlation of IC phosphorylation with dynein association with the total membrane fraction observed when Trk containing signaling endosomes were purified in neurons [38]. It is most likely due to the relatively small contribution that EGFR signaling endosomes make to this total membrane fraction, and we further found that in REF52 cells the high speed pellet material fraction contained only a small (and variable) proportion of total EGF receptor, the majority of which fractionated in the low speed pellet. Unfortunately, our inability to consistently purify the EGF receptor-containing organelles from REF52 cells obstructed our efforts to test the hypothesis further by characterizing this class of organelles (data not shown). It thus remains to be shown if S81 phosphorylation is the general mechanism for recruitment of dynein to receptor signaling endosomes.

### 3.5. Serine 81 Phosphorylation Is without Effect on p150^Glued^ Binding

Dynactin is thought to be important in dynein binding to many cargos (reviewed in [60]). This interaction is mediated by the p150^Glued^ subunit of dynactin which binds directly to the *N*-terminus of dynein intermediate chain. Previously reported sites of dynein phosphorylation (Serine 84 and Threonine 89) proximal to the p150^Glued^ binding site are reported to inhibit the IC-2/p150^Glued^ interaction in gel overlay assays [32,33] and indeed exogenous expression of the S84D phosphomimetic mutant alters Golgi organization [32,61]. Interestingly however, the S84D mutation has a variable effect on the distribution of late endosomes [32,61] indicating that undiscovered regulatory inputs control other dynein-organelle interactions. Given its proximity to Serine 84, Threonine 89 and the p150^Glued^ binding site, we reasoned that Serine 81 phosphorylation might also modulate p150^Glued^ binding. Since gel overlay and solution binding assays have yielded contradictory results [32,61], we chose to use solution binding assays which are more likely to resemble binding conditions in the cytoplasm. Surprisingly, we found no evidence that wild type and phospho-deficient (S81A) IC-2C differed in their ability to co-immunoprecipitate p150^Glued^ from the high speed pellet material of malignant melanoma cells exhibiting constitutive ERK-dependent phosphorylation of IC-2C. Similarly, recombinant (dephospho) wild type and phosphomimetic S81D IC-2C did not detectably differ in their binding to p150^Glued^ in pull-down assays. These observations are in agreement with those of Vaughan and colleagues who, before our formal demonstration of phosphorylation of Serine 81, also found an S81D mutant to be without effect on p150^Glued^ binding in gel overlay assays [32]. While it is possible that the aspartate substitution fails to fully mimic phosphorylation (as is the case for the T89D mutant [33]), together these data suggest that Serine 81 phosphorylation does not regulate p150^Glued^ binding to IC-2C. We hypothesize that Serine 81 phosphorylation might recruit dynein to receptor signaling endosomes by engaging a novel cargo adapter, much like Threonine 89 phosphorylation promotes dynein binding to kinetochores via zw10 [33].

### 3.6. Is Serine 81 Phosphorylation a Universal and Selective Mechanism for Receptor Tyrosine Kinase Trafficking?

The retrograde movement of Trk-containing endosomes is important for axonal and neuronal survival [10]. Trk receptor activity has been shown to be required for this movement, and we recently demonstrated that neurotrophin stimulation of neurons results in MEK-dependent phosphorylation of dynein intermediate chains 1 and 2 on Serine 81 [38]. ERK phosphorylated intermediate chains preferentially associate with Trk-and Rab7-containing organelles, but not with mitochondria. Importantly, expression of non-phosphorylatable intermediate chain 1B reduced nerve growth factor (NGF)-dependent survival of sympathetic neurons, establishing that dynein phosphorylation is not only important for binding to Trk-containing organelles, but for their retrograde Trk transport. Similarly, the retrograde transport of EGF receptors is important for EGF-stimulated mitogenesis in fibroblasts [8]; dynein function is required for perinuclear localization of EGF receptor-containing organelles [12]; and knock-out or inhibition of components of the ERK signaling pathway also inhibit appropriate localization of EGF receptor containing organelles [20,21], Our data now show that EGF, like NGF and brain-derived neurotrophic factor (BDNF), stimulates dynein intermediate chain phosphorylation on Serine 81, although technical limitations prevented us from ascertaining whether Serine 81-phosphorylated intermediate chains preferentially associated with EGF receptor containing organelles. Of importance, Serine 81 phosphorylation enhances binding of dynein to Trk- and Rab7-containing organelles but is without detectable effect on binding to p150^Glued^, implying that Serine 81 phosphorylated intermediate chains bind to a distinct cargo adapter(s) on receptor signaling organelles destined for retrograde transport.

It will be important to determine if other receptor tyrosine kinases, and other classes of cell surface receptor that trigger ERK signaling, also utilize Serine 81 phosphorylation as a means to modify dynein. While EGF, BDNF and NGF are currently the only extracellular stimuli known to cause Serine 81 phosphorylation, our data are consistent with the idea that these receptors utilize Serine 81 phosphorylation as a means to target dynein intermediate chains to receptor tyrosine kinase-containing organelles, perhaps pointing to a universal mechanism for engaging receptor signaling organelles destined for retrograde transport by the dynein motor complex. Moreover, since these receptor tyrosine kinases continue to signal from endosomes on their intracellular journey, and it is known that the amplitude and kinetics of ERK activity can trigger different cell fate outcomes (for example, EGF *vs.* NGF in PC12 cells [62]), it is tempting to speculate that the duration of *in situ* ERK activation and dynein phosphorylation could determine the journey time and/or destination of such receptor signaling endosomes.

While additional studies are clearly necessary to further discern the role of dynamic Serine 81 phosphorylation in regulation of dynein function, the methodology described here is of general utility in identifying novel substrates for ERK in diverse contexts.

## 4. Materials and Methods

### 4.1. Cells

MEK1-null fibroblasts [63] reconstituted with physiological levels of MEK1 (WT cells) or MEK1 mutants will be described elsewhere (Pullikuth and Catling, unsubmitted). REF52 rat embryo fibroblasts were obtained from Dr. Tom Parsons (University of Virginia) and cultured as described [64]. A375 malignant melanoma cells were obtained from ATCC.

### 4.2. Plasmids

mRFP-tagged IC-2C constructs are described elsewhere [38]. Polyhistidine tagged IC-2C in pET21a was a kind gift from Dr. K. Vaughan. The S81D mutation was subcloned using standard methods.

### 4.3. Phospho-Specific Antisera

Generation of the phospho Serine 81 IC-2C antiserum is described elsewhere [38]. We found that the phospho Serine 81 antibody was difficult to strip from membranes (see for instance Figure 4). Hence, we typically ran duplicate membranes for parallel incubation with either phospho Serine 81 antibody or anti-intermediate chain 74.1 antibody.

### 4.4. Anti-Phospho PXSP and PXTP Motif Immunoprecipitation

Novel ERK substrates were identified under license using Cell Signaling Technology, Inc.’s PTMScan technology (Cell Signaling Technology, Inc., Immunoaffinity isolation of modified peptides from complex mixtures, U.S. Patent #7198896, 3^rd^ April 2007 [65]; Cell Signaling Technology, Inc., Immunoaffinity isolation of modified peptides from complex mixtures, U.S. Patent #7300753, 27^th^ November 2007 [66]). WT cells at 80% confluency were washed twice with PBS and serum starved in 0.1% FBS for 16–18 h. Cells were pretreated with or without UO126 (25 μM, Calbiochem) for 3 h and stimulated with EGF (10 ng/mL, Invitrogen, Carlsbad, CA, USA) for 30 min. Cultures were lysed in ice cold lysis buffer (20 mM HEPES-KOH, pH 7.5, 150 mM NaCl, 5 mM MgCl_2_, 1% Trition X-100, 1 mM PMSF, 3 mM benzamidine, 10 μg/mL each of leupeptin and pepstatin, 10 nM microcystin LR, 1 mM sodium orthovanadate, 5 mM sodium pyrophosphate, 50 mM sodium fluoride), and centrifuged at 13,000*g* for 5 min. Cell lysates were precleared with 40 μL of rProtein-A Sepharose CL4B (Invitrogen, Carlsbad, CA, USA) for 1 h at 4 °C, and centrifuged at 600*g* for 5 min. Cleared supernatant was assayed for protein concentration with BCA kit (Pierce, Rockford, IL, USA). Two micrograms protein (in 1 mL volume) was incubated with anti-PXSP (15 μL) or anti-PXTP (15 μL, both from Lot 2, Cell Signaling Technology, Beverly, MA, USA) overnight at 4 °C. Immune complexes were sedimented with 40 μL of rProtein-A Sepharose CL4B for 2 h at 4 °C followed by two washes in lysis buffer (1 mL each time). Beads were suspended in 100 μL 2X SDS-Sample buffer, boiled, centrifuged and the supernatant was assayed by western blotting with anti-dynein intermediate chain mAb (Clone 74.1 [27]; Covance, Princeton, NJ, USA). For DIGE labeling, immune complexes were subsequently washed twice with water and processed as described below.

### 4.5. Identification of Candidate ERK Substrates

Anti-phospho PXSP immunoprecipitates from control and UO126-treated cells were suspended in 7 M urea, 2 M thiourea, 4% CHAPS, 20% glycerol, 30 mM Tris, pH 8.5, prior to labeling with 400 pmol either Cy3 (DMSO/EGF) or Cy5 (UO126/EGF) for 30 min. on ice in the dark. Reactions were quenched by the addition of 10 mM lysine for 10 min. on ice in the dark. This protocol results in minimal labeling (~1%) to minimize molecular weight shifting and results in sensitivities on the order ~1 ng. The Cy3- and Cy5-labeled samples were combined, mixed with an equal volume of Destreak Rehydration Buffer (GE Healthcare, Pittsburgh, PA, USA) and actively rehydrated into 24 cm 3–10 nL immobilized pH gradient (IPG) strips (GE Healthcare) for 15 h, followed by isoelectric focusing using an Ettan IPGphor II (GE Healthcare) for a total of 90,000 Vhrs (step 300V 1 h, gradient 1000 V 6 h, gradient 8000 V for 6 h, step 8000 V for 8 h and a final step 250 V for HOLD). Cysteines were reduced and carbamidomethylated while the proteins were equilibrated into the second-dimensional loading buffer by incubating the focused strips in equilibration buffer (6 M urea, 20% glycerol, 2% SDS, 375 mM tris, pH 8.8) supplemented with 20 mg/mL DTT for 15 min. at room temperature with shaking, followed by 25 mg/mL iodoacetamide in equilibration buffer for an additional 15 min. room temperature incubation. IPG strips are then cemented onto 2^nd^ dimension gels using an overlay consisting of 0.5% agarose in SDS running buffer (25 mM Tris, 192 mM glycine, 0.1% SDS, trace of Bromophenol Blue). Second-dimension SDS-10% PAGE was performed using a DALT*six* system (GE Healthcare, Pittsburgh, PA, USA) at 5 W/gel for 30 min. followed by 13 W/gel for 4 h at 25 °C. The Cy3 and Cy5 signals for each gel were individually imaged on a Typhoon 9400 scanner (GE Healthcare, Pittsburgh, PA, USA) using mutually exclusive excitation/emission wavelengths of 532nm (ex) and 580nm (em) for Cy3, and 633nm (ex) and 670 nm (em) for Cy5. After imaging, the gels were fixed (10% methanol, 7% acetic acid; 1 h), rinsed in water three times and then incubated overnight in SYPRO Ruby in the dark. The SYPRO Ruby post-stain allows for the correction of the unlabeled proteins’ migration in relation to the cognate Cy-Dye labeled proteins, and ensures accurate spot excision. SYPRO Ruby images were acquired on the Typhoon using 450 nm (ex) and 610 nm (em) filters, and re-imaged post-excision to ensure accurate spot picking. Proteins whose Cy5 fluorescence was decreased (*i.e.*, whose immunoprecipitation with antiphospho PXSP antiserum was decreased following UO126 treatment) were identified using DeCyder software (GE Healthcare, Pittsburgh, PA, USA) and picked using a robotic Ettan Spot Handling Workstation (GE Healthcare, Pittsburgh, PA, USA). Spots were de-stained by successive changes of 20 mM ammonium bicarbonate and 50% acetonitrile, followed by dehydration with 100% acetonitrile (20 min.). Dehydrated gel plugs were digested in-gel with 8 μL 20 μg/mL porcine modified trypsin protease (Promega) in 20 mM ammonium bicarbonate for 6 h at 37 °C. Tryptic peptides were then extracted from the gel plugs in two cycles of 50% acetonitrile/0.1% trifluoroacetic acid and dried by evaporation. Peptides were reconstituted in 5 μL 2% acetonitrile/0.1% formic acid prior to LC-MS analysis. A Thermo LTQ-XL (CA) was coupled with an Eskigent nanoLC system (Framingham, MA, USA). Peptides were loaded on a Dionex C18 trap column and separated on a PicoFrit C18 column/emitter at 200 nL/min with gradient 2% acetonitrile/0.1% formic acid to 40% acetonitrile/0.1% formic acid in 20 min., to 60% acteonitrile in 5 min, to 90% acetonitrile in 15 min. before returning to starting solvent. The peptides are eluted directly into the LTQ-XL mass spectrometer. One survey scan was acquired first. The 5 most abundant peptide ions (precursor ions) were selected for MS/MS for partial sequencing. In-house MASCOT 2.2 (Matrix Science, London, UK) was used to search against SWISS-PROT and NCBI non-redundant rodent databases (updated 6/2009) without constraining protein molecular weight or isoelectric point and allowing for carbamidomethylation of cysteine, partial oxidation of methionine residues, and one missed trypsin cleavage. The significance threshold of 5% probability (Mascot default) was used. Proteins with protein scores above 55 and two or more peptides with ion scores greater than 20 are considered matches.

### 4.6. Identification of Phosphorylation Sites in Dynein Intermediate Chain 2C

Confluent 15 cm plates of WT cells (2 per time point) were serum deprived overnight in DMEM/0.1% FBS before incubation with or without 10 ng/mL EGF for 30 min. at 37 °C. Lysates were prepared in RIPA buffer containing protease and phosphatases inhibitors, clarified by centrifugation and triplicate 1.7 mg aliquots of lysate protein were immunoprecipitated with 10 μg anti-dynein intermediate chain antibody 74.1 [27] for 3 h at 4 °C. The mixture was re-centrifuged and the supernatant transferred to a clean tube containing protein A agarose. After 1 h at 4 °C, the immunoprecipitates were washed three times with lysis buffer before electrophoresis on an 8% gel. The gel was fixed in three changes of 10% acetic acid/50% methanol over 2 h, and stained with Coomassie Blue. After destaining, IC-2C bands were excised, transferred to a siliconized tube, washed and destained in 200 μL 50% methanol overnight. The gel pieces were dehydrated in acetonitrile, rehydrated in 30 μL of 10 mM DTT in 0.1 M ammonium bicarbonate and reduced at room temperature for 0.5 h. The DTT solution was removed and the sample was alkylated in 30 μL 50 mM iodoacetamide in 0.1 M ammonium bicarbonate at room temperature for 0.5 h. The reagent was removed and the gel pieces dehydrated in 100 μL acetonitrile. The acetonitrile was removed and the gel pieces rehydrated in 100 μL 0.1 M ammonium bicarbonate. The pieces were dehydrated in 100 μL acetonitrile, the acetonitrile was removed and the pieces completely dried by vacuum centrifugation. The gel pieces were rehydrated in 20 ng/μL of Trypsin, endoproteinase Glu-C or endoproteinase Asp-N in 50 mM ammonium bicarbonate on ice for 10 min. Any excess enzyme solution was removed and 20 μL 50 mM ammonium bicarbonate added. The sample was digested overnight at 37 °C and extracted in two 30 μL aliquots of 50% acetonitrile/5% formic acid. These extracts were combined and evaporated to 15 μL for mass spectrometry analysis.

The LC-MS system consisted of a Thermo Electron LTQFT (Surveyor HPLC) mass spectrometer system with a Protana nanospray ion source interfaced to a self-packed 8 cm × 75 um i.d. Phenomenex Jupiter 10 μm C_18_ reversed-phase capillary column. 7.5 μL volumes of the extracts were injected and the peptides eluted from the column by an acetonitrile/0.1 M acetic acid gradient at a flow rate of 0.5 μL/min over 1 h. The nanospray ion source was operated at 2.5 kV. The digest was analyzed by acquiring 1 full scan mass spectra (MS-100K, ICR) to determine peptide molecular weights followed by 10 product ion spectra (MS/MS-ion trap) to determine amino acid sequences. The data were analyzed by using the Sequest search algorithm (Bioworks 3.3.1) against mouse IC-2C. Any potentially phosphorylated peptides that passed minimal cutoff scores and 10ppm parent mass error were manually verified for identity and modification site. Once the sequence and modification site were verified, the data was further examined to determine all possible forms of each peptide that could be identified in each time point digest—nonphosphorylated, phosphorylated, missed cleavages, oxidized Met, acrylamide Cys, *etc.* Areas (phosphorylated and nonphosphorylated) for all forms of a peptide were determined using selected ion chromatograms from QualBrowser (Xcalibur 2.1) using the most abundant isotope with a mass window of +/− 0.02Da. A ratio of phosphorylated divided by nonphosphorylated was determined for each sample. Semi-quantitative changes in extent of phosphorylation were determined by comparing this ratio in the EGF-stimulated sample to the unstimulated sample.

### 4.7. Subcellular Fractionation

Isolation of membrane-bound organelles was accomplished essentially as described [44,45]. Briefly, REF52 cells were serum-deprived overnight before treatment with or without EGF (10 ng/mL) for the indicated times. In some experiments UO126 was added prior to growth factor stimulation. Cultures were washed twice in PBS and once in cytoplasm-like buffer (CB, 38 mM each of the potassium salts of aspartic, gluconic, and glutamic acids; 20 mM MOPS; 5 mM reduced glutathione; 10 mM potassium bicarbonate; 0.5 mM magnesium carbonate; 1 mM EGTA; 1 mM EDTA adjusted to pH 7.1 at 37 °C with potassium hydroxide). All procedures were carried out on ice or at 4 °C. Cells were scraped into CB, gently pelleted and resuspended in CB containing protease and phosphatases inhibitors. After homogenization by 8 passes through a ball bearing homogenizer (Isobiotec; 8 micron clearance) extract was centrifuged twice at 1000*g* for 10 min. to yield low speed supernatant (S1) and pellet (P1) fractions. The S1 fraction was subsequently centrifuged at 100,000*g* for 60 min. to yield the high speed supernatant (S2) and pellet (P2) fractions. Portions equivalent to a fixed number of cells were resolved on 8%–15% gradient gels.

### 4.8. Recombinant Proteins and Pull down Assays

*E. coli* Rosetta (DE3) pLysS (Novagen, Madison, WI, USA) harboring rat dynein intermediate chain 2C in pET 21a were grown to *OD*_600_ = 0.6 in 3L LB media at 37 °C. Cultures were cooled to 30 °C and induced with 0.3 mM IPTG for 2 h. The following steps were carried out at 0–4 °C unless noted. Cells were lysed in TN buffer (50 mM Tris-HCl, 50 mM NaCl, pH 8.0) containing 15 mM imidazole and protease inhibitors by two passes through an Emulsiflex C5 (Avestin, Ottawa, Canada). Lysates were clarified by centrifugation at 100,000*g* for 60 min, and loaded onto a 1 mL HiTrap HP Nickel column (GE Healthcare, Pittsburgh, PA, USA). The column was washed extensively with TN/15 mM imidazole before elution with a linear gradient of 15–500 mM imidazole in TN. Fractions containing IC-2C (200–300 mM imidazole) were pooled and applied to a Q2 anion exchange column (BioRad, Hercules, CA, USA) equilibrated in TN before elution with a linear gradient of 0–450 mM NaCl in TN. Fractions containing IC-2C were pooled, frozen as aliquots in liquid nitrogen and stored at −80 °C. Yield was approximately 0.5–1 mg per 3 L culture, of which approximately 50%–75% was full length IC-2C protein. To determine if phosphorylation of Serine 81 modulates binding of IC-2C to p150^Glued^ 0, 0.5, 2.5 and 12.5 ug of recombinant polyhistidine-tagged wild type or S81D IC-2C were incubated with rat brain lysate overnight, subsequently captured with Nickel agarose and extensively washed with FLAG buffer [67]. Bound proteins were eluted with 1 M imidazole in TN buffer for 1 h on ice and blotted with anti-p150^Glued^ antiserum.

### 4.9. Co-Immunoprecipitation Assays

P2 fractions were prepared from A375 melanoma cells transfected with mRFP-tagged IC-2C constructs. Pellets were resuspended in cold CB buffer and immunoprecipitated with monoclonal anti-RFP antibody (MBL, Madison, WI, USA) for 2.5 h at 4 °C. Immune complexes were bound to protein G-coupled Dynabeads (Invitrogen, Carlsbad, CA, USA) and collected with a magnetic tube rack according to manufacturer’s instructions. After washing 3 times with cold CB buffer, bound proteins were eluted with 200 mM glycine, pH 2 for 30 min. on ice and blotted with anti-RFP (Abcam, Cambridge, MA, USA) and anti-p150^Glued^ antisera (BD Biosciences, San Jose, CA, USA).

## Figures and Tables

**Table 1:** Identification of six phosphorylation sites in IC-2 from EGF-stimulated cells.

Protease Digest	IC-2C peptide	+P/−P ratio, EGF 30 min
Trypsin	EAEALLQSMGLTTDSPIVPPPMSPSSK (S81)	4.873
Endo AspN	DSPIVPPPMSPSSKSVSTPSEAGSQ (S81)	3.318
Trypsin	SVSTPSEAGSQDSGDGAVGSR (T89/S95/S98 *)	0.006
Endo AspN	DDVATPKPPVEPEEEKTLKKDEEN (T154)	0.080
Endo GluC	TQTPVTAQPKEDEEEEDDVATPKPPVEPEEE (T154)	0.019
Endo GluC	AAVSVQE (S51)	0.005

Phosphopeptides were identified by mass spectrometry as described in Materials and Methods. The abundance of the phospho- (+P) and corresponding non-phosphopeptides (−P) was determined following 30 min. EGF stimulation.

* Phosphoisomers of this peptide were incompletely resolved by chromatography and hence they are considered as a group.

**Table 2:** Identification of Serine 81 phosphorylation after Trypsin or endo Asp-N digestion.

Protease	Digest IC-2C S81 peptide		Control	EGF
Trypsin				
	EAE….		1.74 × 10^6^	1.45 × 10^6^
	EAEmM….		1.39 × 10^6^	8.57 × 10^6^
	EAEMm….		2.68 × 10^6^	2.58 × 10^6^
	EAEmm….		5.62 × 10^6^	4.75 × 10^6^
		Total	1.14 × 10^7^	9.64 × 10^6^
	EAE p….		1.60 × 10^6^	8.66 × 10^6^
	EAEmM p….		1.21 × 10^6^	5.50 × 10^6^
	EAEMm p….		2.35 × 10^6^	1.22 × 10^6^
	EAEmm p….		4.10 × 10^6^	2.06 × 10^6^
		Total	9.26 × 10^6^	4.70 × 10^7^
		+P/−P	0.81	4.87
		Fold change	1	6.01
Endo Asp-N				
	DSP….		1.67 × 10^7^	1.61 × 10^6^
	DSPm….		2.98 × 10^7^	9.66 × 10^6^
		Total	4.65 × 10^7^	1.13 × 10^7^
	DSP p….		8.27 × 10^6^	7.69 × 10^6^
	DSPm p….		1.97 × 10^7^	2.97 × 10^7^
		Total	2.80 × 10^7^	3.74 × 10^7^
		+P/−P	0.60	3.32
		Fold change	1	5.52

The abundance of peptides containing phospho- and non-phospho Serine 81 was determined. M and m methionine and oxidized methionine residues respectively; since the Tryptic peptide containing Serine 81 contains two methionine residues, four peptide masses are possible for the phosphorylated (p) species, and four are possible for the non-phospho species. The ratio of the abundance of the phospho and non-phospho forms was then compared between control and EGF treatment conditions to give an approximate fold induction in phosphorylation.
